# Accessing maternal and child health services in Melbourne, Australia: Reflections from refugee families and service providers

**DOI:** 10.1186/1472-6963-12-117

**Published:** 2012-05-15

**Authors:** Elisha Riggs, Elise Davis, Lisa Gibbs, Karen Block, Jo Szwarc, Sue Casey, Philippa Duell-Piening, Elizabeth Waters

**Affiliations:** 1 The Jack Brockhoff Child Health and Wellbeing Program, The McCaughey Centre, The University of Melbourne, Melbourne, Australia; 2 Healthy Mothers Healthy Families Research Group, Murdoch Childrens Research Institute, Melbourne, Australia; 3 The Victorian Foundation for the Survivors of Torture (Foundation House), Melbourne, Australia

**Keywords:** Refugee, Maternal and child health, Access to health services, Cultural competence

## Abstract

**Background:**

Often new arrivals from refugee backgrounds have experienced poor health and limited access to healthcare services. The maternal and child health (MCH) service in Victoria, Australia, is a joint local and state government operated, cost-free service available to all mothers of children aged 0–6 years. Although well-child healthcare visits are useful in identifying health issues early, there has been limited investigation in the use of these services for families from refugee backgrounds. This study aims to explore experiences of using MCH services, from the perspective of families from refugee backgrounds and service providers.

**Methods:**

We used a qualitative study design informed by the socioecological model of health and a cultural competence approach. Two geographical areas of Melbourne were selected to invite participants. Seven focus groups were conducted with 87 mothers from Karen, Iraqi, Assyrian Chaldean, Lebanese, South Sudanese and Bhutanese backgrounds, who had lived an average of 4.7 years in Australia (range one month-18 years). Participants had a total of 249 children, of these 150 were born in Australia. Four focus groups and five interviews were conducted with MCH nurses, other healthcare providers and bicultural workers.

**Results:**

Four themes were identified: facilitating access to MCH services; promoting continued engagement with the MCH service; language challenges; and what is working well and could be done better. Several processes were identified that facilitated initial access to the MCH service but there were implications for continued use of the service. The MCH service was not formally notified of new parents arriving with young children. Pre-arranged group appointments by MCH nurses for parents who attended playgroups worked well to increase ongoing service engagement. Barriers for parents in using MCH services included access to transportation, lack of confidence in speaking English and making phone bookings. Service users and providers reported that continuity of nurse and interpreter is preferred for increasing client-provider trust and ongoing engagement.

**Conclusions:**

Although participants who had children born in Melbourne had good initial access to, and experience of, using MCH services, significant barriers remain. A systems-oriented, culturally competent approach to service provision would improve the service utilisation experience for parents and providers, including formalising links and notifications between settlement services and MCH services.

## Background

Australia currently accepts approximately 13,750 refugees per year with approximately 4,000 settling in the state of Victoria [1]. Many new arrivals from refugee backgrounds have experienced poor health and limited access to healthcare services [2]. Families from refugee backgrounds face a range of challenges that can affect child rearing practices, due to the experience of torture and trauma, changes in family roles, separation of family members and poor access to primary healthcare [3,4]. Traditional support networks are often missing due to loss and separation of family and community. Families from refugee backgrounds may have faced extreme circumstances which reduce their capacity to adapt to their new environment. Settlement difficulties may be exacerbated by unemployment, financial instability and financial responsibility and concern for family members who remain overseas or in refugee camps [3,5]. Access to health services can be difficult for families with children of refugee backgrounds because of a lack of culturally appropriate information, cultural differences in practices such as child rearing, as well as a limited understanding of Australia’s health system [6], which could be addressed by a culturally competent healthcare system that is responsive to these issues. Gender, education, occupation, income and ethnicity and place of residence are all closely linked to people’s access to, experiences of, and benefits from health care [7]. In addition, because refugee children and families may present with a range of health issues which are unfamiliar to Australian healthcare professionals [6], their complex needs may go undetected.

### Victorian maternal and child health service

In Australia, the Victorian maternal and child health (MCH) service is a longstanding state-wide government operated, universal, primary healthcare system for families with children under six years delivered in partnership between state and local government [8]. The law in Victoria prescribes that the notice of a birth must be given by the responsible person (usually the hospital) to the council of the municipal district in which the mother of the child usually resides [9]. On receipt of the birth notice the council must, send a copy of the notice to the MCH nurse whose duty it is to contact and visit that household. New mothers then receive a home visit from a MCH nurse. All consultations thereafter usually require the family to visit the centre for the remaining 9 of the 10‘key ages and stages’ aligning with the child’s age at: 2, 4, 8 weeks; 4, 8, 12 and 18 months; 2 and 3.5 years. These consultations involve the MCH nurse conducting assessments of the child and discuss any of the mother’s concerns about their own health. The service offers:

· support, information and access to professional advice on everything from child behaviour and nutrition to breastfeeding and family planning

· the opportunity to identify any problems in a child’s health and development

· the chance to meet other local parents and access parent and community groups

· further support, referrals and assistance for parents who need it [10].

If a family moves from their municipality and proactively reconnects with the MCH service in their new area, the MCH service has an administrative process that seeks the client’s records from the previous MCH centre. However, there is the potential, for families to be ‘lost’ to the MCH service following a move as the onus is on the family to reconnect with the service.

Prevention programs which identify and address problems early have been shown to dramatically save costs compared with later interventions that focus on treatment [11]. Universal systems are intended to provide ready access to all potential users and delays in the early detection of illness or developmental issues increases the likelihood that children remain vulnerable and at heightened risk for experiencing problems as they enter the education system [12]. A recent review of government early childhood services found that although access to universal services for vulnerable children has improved over the past five years, indicated by increasing participation rates, the Department of Education and Early Childhood Development (DEECD) cannot demonstrate that these services are accessible when and where needed, especially for vulnerable children and families. The report concluded:

"*The Department’s inability to reliably identify all vulnerable children and families means that is does not know the extent to which children are missing out on the benefits of attending targeted services specifically developed and funded to meet their needs*[12]*.*"

For new arrivals to Victoria, awareness of the MCH service can be challenging as there is no formal mechanism for MCH nurses to be notified of these families [13]. Findings from a recent evaluation of the service suggest that MCH services initially engage culturally and linguistically diverse (CALD) and single and/or young mothers well, but subsequent disengagement results in reduced levels of service utilisation and satisfaction [14]. While CALD participants were included in the evaluation, the report does not define who are included in the CALD sample and whether this includes refugee background participants. Approximately 80% of CALD mothers received the first home visit, a few days after birth and this reduced to approximately 35% making the 3.5 year visit, compared with Australian-born mothers (not including the Indigenous population) of whom approximately 95% and 65% receive and make these visits respectively [14]. The authors conclude that language and cultural barriers influence access, resulting in people with lower levels of English feeling less positive about the usefulness of their visit to the MCH and less likely to continue using the service and they suggest that this can be offset by culturally responsive services. A key finding of an extensive literature review outlining the role and nature of universal health services (including MCH services) was the need to better understand the experiences and needs of disadvantaged or vulnerable women, children and families who receive these services [15]; in particular, the factors that facilitate or hinder decisions to accept or access these services.

The MCH service provides an opportunity to focus on the dynamics between parents and children and to locate the issues facing refugees in their wider family and community context. However, a needs assessment of MCH nurse and coordinator needs was conducted and a major finding was the nurses themselves expressed a desire for professional development for working with vulnerable clients (including culturally and linguistically diverse clients) and working in partnership with families [16]. Furthermore, a recent Victorian report examining the health, wellbeing, development, learning and safety of children and young people of refugee backgrounds highlighted a lack of capacity to identify refugee background people and the limitations and inadequacies of available datasets in multiple sectors (for example, maternal and child health, perinatal health and child development) [17].

### Prevention and promotion through culturally competent healthcare

Internationally, a study conducted in Sweden with primary healthcare nurses found that models of healthcare services that promote holistic family-centred approaches to ‘involuntary migrants’ require a range of skills that may not necessarily be incorporated in current professional education and training [18]. This approach requires nurses to cooperate with other health care professionals and community authorities and to practise family-focused nursing; it also demands skills in intercultural communication paired with cultural self-awareness in interacting with families [18]. Kemp and colleagues suggest that to meet the additional and complex needs of families with low English-fluency, service improvements require an increase in workforce numbers, adequate provision of clinical supervision, additional ongoing professional development and planning for future education needs [19]. While there are programs for psychological interventions to address many of the traumatic experiences faced by refugees [20,21], some scholars and practitioners suggest that there is a need for more community-based health promotion approaches to target health needs using community media and social networks [22]. For example, there is evidence that the use of specialist ‘asylum support nurses’ improved access to primary medical care services in the United Kingdom [23]. In Victoria, ‘refugee health nurses’ support comprehensive health assessments and care of newly arrived refugees and assist and refer them to other primary and specialist health services including the MCH service [24]. The refugee health nurse program aims to: increase refugee access to primary health services and improve the response of health services to refugees’ needs. The refugee health nurses play a crucial role in working directly with refugee communities, supporting timely access to health assessment, optimal care co-ordination and advising other health practitioners on refugee health matters. They are located in 16 community health services in areas of significant refugee settlement in metropolitan and rural Victoria.

Cultural competence is a set of congruent behaviours, attitudes and policies that come together in a system, agency, or among professionals and enable effective work in cross-cultural situations [25]. Across the literature, associations between perceptions of racial and/or ethnic discrimination and poor service utilisation in the provision of healthcare services to culturally diverse communities living in developed countries are reported [26]. A culturally competent system may have the potential to reduce racial and ethnic health disparities [27]. A study in the U.S. demonstrated that perceived discrimination is associated with delays in people seeking care and adhering to medical advice [28]. Studies show that active outreach, education and health promotion activities can increase utilisation of healthcare services for communities from refugee backgrounds (specifically, in this case, from Sub Sahara African and Afghani, Iraqi and Nepali backgrounds) [22]. Such strategies include using local ethnic media, as well as active engagement by disseminating health and health services information through personal and community networks [29]. The Australian National Health and Medical Research Council (NHMRC) has previously called for research to be conducted to improve the effectiveness of culturally competent healthcare systems in increasing client satisfaction with care received, enhancing client health and reducing inappropriate racial and ethnic differences in use of health services or in received or recommended treatment [30]. Woodland and colleagues have identified ten elements that provide a practical framework for improving access, equity and quality of care in service delivery for newly arrived refugee children [31]. These include: 1) routine comprehensive health screening; 2) co-ordination of initial and ongoing health care; 3) integration of physical, developmental and psychological health care; 4) consumer participation; 5) culturally and linguistically appropriate service provision; 6) inter-sectoral collaboration; 7) accessible and affordable services and treatments; 8) data collection and evaluation to inform evidence-based practice; 9) capacity building and sustainability and 10) advocacy. The authors suggest that these elements of good practice can be applied to reduce the gap between health needs and the services that are currently available [31].

A socioecological approach attends to the interaction of individual, community, institutional and societal factors affecting health and wellbeing. This approach demonstrates the complexity of the interactions between health, its determinants and its outcomes [32] and can provide insight into dealing with complex health issues as well as addressing basic health problems [33-35]. It highlights the importance of a holistic view of health, including physical, psychological, social and spiritual dimensions. It is generally accepted that an individual’s health is determined by a range of factors, including environmental and economic factors, many of which are outside the direct control of the individual. In some countries, social determinants may include circumstances such as geography, ethnicity and socioeconomic status [36]. These social determinants of health are commonly used within public health and health promotion literature to provide a greater understanding of the equality and equity issues found in health outcomes within communities. The work of the Commission on Social Determinants of Health of the World Health Organisation has significantly advanced understanding of the impacts of social determinants on health outcomes. Of particular pertinence to this research, the Commission’s final report states that ‘investment in the early years provides one of the greatest potentials to reduce health inequities within a generation…that mothers and children need a continuum of care from pre-pregnancy, through pregnancy and childbirth, to the early days and years of life’[37](p4). The Commission accordingly calls for health care systems to be based on principles of equity, disease prevention and health promotion.

### Study rationale

Given that the MCH service is critical for promoting the health and wellbeing of young children and their families, particularly mothers, it is important to examine its utilisation by families from refugee backgrounds. Much of the research conducted to date does not differentiate between people of refugee background and non-refugee immigrants, and/or refers to vulnerable, deprived, disadvantaged and low-income groups. In this paper it is argued that there is a need to better understand how parents of refugee background experience MCH services and how best to support them in accessing these universally available services to promote the health and wellbeing of them and their children. This study aims to explore the utilisation and experience of MCH services in Melbourne, Victoria for parents of refugee background from the perspective of users and providers.

## Methods

### Ethics

Ethics approval was provided by The University of Melbourne and the Department of Education and Early Childhood Development (DEECD).

### Theoretical framework

The study is underpinned by a socioecological model of health [38] i.e. considerations of the social determinants influencing health outcomes, and a cultural competence approach i.e. involvement of community representatives in guiding all stages of the research [39,40].

### Method

Qualitative methods were used as the most appropriate way to explore the lived experiences of participants and to identify the range of facilitators and barriers to service access. Focus groups were conducted with women of refugee backgrounds; this method was supported by community representatives and the participants themselves, as an appropriate approach for data collection. Focus groups were also chosen for data collection from MCH nurses and other healthcare providers. Bilingual community workers and their managers were invited to participate in individual interviews for pragmatic reasons. These individuals worked at different organisations and locations and it was their preference for the interviewer to meet them at their place of employment.

### Selection and recruitment

An advisory group established for the study selected two different geographical areas (Wyndham and Hume) in Melbourne, identified using settlement data [41] as having high numbers of families with young children with a refugee background including Karen/Burmese and Iraqi/Assyrian Chaldean. At the time, these geographic areas were not involved in other research projects. The sample included mothers of refugee backgrounds with children aged 0–6 years, MCH nurses, other health service providers, and community representatives including bilingual workers. Participants were recruited from existing playgroups, a kindergarten, a peer education program, and an adult English language organisation. Participants were invited to participate through a bilingual worker or health worker that was known to them. Participants were provided with a plain language statement and consent form available in English, Karen and Arabic languages (due to the late addition of the Bhutanese participants the forms were read aloud by a Nepali interpreter as there was not enough time to allow for translating).

Participants included in the initial focus groups were not necessarily representative of all newly-arrived refugee mothers as almost all were in full-time child rearing roles and were not learning English in formal programs. Concurrent data analysis indicated that there were different access experiences for different groups depending on whether they were attending English classes were caring for children full-time. Therefore further sampling was required to access other cultural groups including: South Sudanese, Bhutanese and also more Iraqi women who were studying English. This was necessary to deepen the understanding of the range and depth of experiences that parents of refugee background have engaging with the MCH system. Purposive sampling was also used to invite MCH nurses and other healthcare service providers to participate in the study. They were sought to provide data on their experiences of working with clients of refugee backgrounds and perceived barriers for continuous engagement with MCH services. Bilingual community workers were also invited to participate in the study as they were identified as often critical for facilitating access to MCH services. Parents received a $30 supermarket shopping voucher to thank them for participating.

### Data collection and analysis

Seven focus groups with parents were conducted in a mixture of first language spoken and English. Four of the focus groups were interpreted by a bilingual community worker and three were interpreted by an accredited interpreter. All focus groups and interviews were conducted by ER and either KB or ED was present to assist with note taking. ER kept detailed field notes of meetings with other stakeholders to provide further insight. All focus groups and interviews were digitally recorded the English content was transcribed, including the interpreter translations. Focus group and interview guides were developed by the advisory group with feedback from the bicultural workers regarding cultural appropriateness. The questions explored information relating to access to MCH service as well as other health professionals and services, who and where information was sought regarding child health and development and suggested improvements for the MCH service Examples of questions for mothers included ‘Have you ever used the maternal and child health service here in Melbourne?’ and ‘Is there anything that prevents you from making an appointment to see the maternal and child health nurse?’ The managers of the MCH nurses also reviewed the questions to be asked of the nurses to ensure they were relevant for their staff and that participation in the research would be useful to them. The wording of the questions for the bicultural community workers was modified by the researchers to ensure they were relevant in terms of their role with the cultural community that they typically worked with. The questions covered the same topics but were phrased as ‘From your perspective, what are the main barriers for families of refugee backgrounds to access MCH services?’

A process of thematic analysis was used. ER listened to all voice recordings, read and coded all transcripts, and developed categories to organise the data. ED and LG also read a sub-sample of transcripts and coded them. The coding was found to be very similar with any differences discussed by the researchers to arrive at a consensus about final codes. The researchers also discussed patterns, consistencies and contradictions within the data to develop the main themes. ER then refined the themes in consideration of their alignment with the existing literature. All research investigators and the study advisory group came together to discuss the themes, further interpret and explain the results and the implications and applications of the findings.

Some qualitative scholars would argue that the data gathered from an individual interview is different from the data gathered in a focus group and that they ‘defy direct comparison’ [42]. However, combining focus group and individual interview data can be advantageous as complementary views of the topic being explored may be generated. Lambert and Loiselle identify three broad rationales for combining such data for analysis: 1) pragmatic reasons (that is, those who cannot or choose not to attend the focus group); 2) the need to compare and contrast participants’ perspectives (parallel use); and 3) striving toward data completeness and/or confirmation (integrated use) [43]. Given that the aim of this research was to gain a deeper understanding of the issue at hand and to develop a cohesive set of recommendations, it made sense to combine the data produced from the two methods. Lambert and Loiselle argue that combining focus group and interview data enhances data richness and that combining different qualitative data sources is a form of triangulation [43]. Explicitly recognising and acknowledging the impact of the combination of method and data is a productive strategy that can lead to an enhanced description of the phenomenon being explored.

## Results

Seven focus groups were conducted with 87 parents, all female (Table 1). Participants were from Karen, Iraqi, Assyrian Chaldean, Lebanese, South Sudanese and Bhutanese cultural backgrounds, who had lived an average of 4.7 years in Australia (range one month-18 years). Participants had a total of 249 children. Of these, 150 were born in Australia, with one to seven children per family.

**Table 1:** Participant characteristics

**Cultural background of participants**	**Site of recruitment**	**No. of participants**	**Mean time in Aus. (yrs)**	**No. of children (A: born in Australia, O: born overseas)**	**Who was the interpreter for focus group?**
Karen	Playgroup organised by refugee mentor	14	5.6	A: 15	Bilingual refugee mentor (also an accredited interpreter)
Burmese				O: 26	
Karen	Playgroup organised by refugee mentor	29	5.5	A: 32	Bilingual refugee mentor (also an accredited interpreter)
Burmese				O: 50	
Karen	Kindergarten	4	3.3	A: 4	Accredited Interpreter
Burmese				O: 7	
Iraqi	Playgroup organised by refugee mentor	18	8.5	A: 38	Bilingual refugee mentor
Assyrian Chaldean				O: 12	
Iraqi	Studying English at an adult English language centre organised by an cultural welfare agency	8	4.3	A: 11	Bilingual community representative
Assyrian Chaldean				O: 11	
Lebanese					
South Sudanese	Playgroup organised by refugee mentor	7	6.3	A: 16	Bilingual refugee mentor
				O: 17	
Bhutanese	Peer education program at community health service	7	1.6	A: 4	Accredited Interpreter
				O: 6	

Five interviews and four focus groups were also held with a total of 18 service providers and bilingual workers as follows: Two focus groups were conducted with three and four MCH nurses in each. Another two focus groups were held with two refugee health nurses in each. One of these focus groups also included another MCH nurse together with a worker from the ‘Healthy Mothers Healthy Babies’ program and the other focus group included an Arabic community liaison worker. The objectives of the Healthy Mothers, Healthy Babies Program are to support and provide assistance to women to access antenatal, postnatal and other health and human services throughout their pregnancy [44]. Three interviews were conducted with community representatives/refugee mentors (who were also the bilingual playgroup facilitators). Two interviews were conducted with managers of bilingual workers (one of whom is also a bilingual community worker).

Four main themes were identified, these are: ‘Facilitating access to MCH services’; ‘Promoting continued engagement with the MCH service’; ‘Language challenges,’ and; ‘What is working well and what can be done better’. These are reported here with their subthemes.

### Facilitating access to MCH services

There were four main modes identified for facilitating initial access to the MCH service. These included: the birth notification service, by settlement case workers, by refugee health nurses or by bicultural playgroup facilitators (refugee mentors).

### Birth notification from hospital

Participants who had given birth in a local hospital were contacted by the MCH service via the birth notification system and received a home visit. Most mothers felt that this process worked well for them. In one focus group, the mothers reported that it was much easier for those who had children born in Australia because they are able to learn the ‘*new system’*. The mothers with children born outside Australia, even those with good grasp of spoken English, were still trying to catch-up and learn the way in which the healthcare services operated.

"*I think it is good for her [another mother in the playgroup] because she started having children here and she has a lot of support. I am a mother of 7 kids, I’m only here, my husband went back to Sudan and I’m working and I got other thing to do…it’s a big change for us…this life for me is hard… if they can give us a special way, they can call us and say you didn’t come to check which time is good for you to come, you know?… I know anyway it’s my job to [take children to see the MCH nurse] because it’s my child and it’s important for me to care about their health but the situation I’m in, it’s hard…(South Sudanese FG)*"

### Settlement case workers

There was no consistent model identified for introduction to the MCH service for parents who arrived in Melbourne with young children. Some families were linked to the MCH service by their case-worker/community guide who is provided to them in the first six months of arrival by a government funded settlement service. However, at the time of the study, not all refugee-background families were eligible for this service. People of refugee background accepted into Australia as part of the humanitarian migration stream may arrive on a refugee visa making them eligible for the full range of settlement services. Others receive a special humanitarian visa provided to those with family members already here, in which case their ‘sponsoring’ family members were expected to facilitate their access to services. The healthcare professionals reported that many people they saw on ‘sponsored’ visas had not been linked to the service. In many cases ‘sponsors’ were also in need of services and support and were not in a position to be primarily responsible for facilitating service access for others. (Note: since the time of data collection, the policy regarding this type of visa has been changed so that now those who arrive on a ‘sponsored’ visa receive the full suite of settlement services support). The participants who had a community worker reported that it is likely that even if they were told about the MCH service in their first six months of settlement, it is likely that they forgot about it, as there were other settlement priorities such as housing, employment and learning English that took precedence.

### Refugee health nurses

The refugee health nurses reported that they identified people in need of the MCH service when conducting home visits as part of the ‘refugee health assessment’ and they facilitated making an appointment for them. However, it was reported by several of the refugee health nurses that the refugee health nurse program did not have the capacity to identify and meet the demand of all newly-arrived parents and children. This indicates that there are likely to be parents and children arriving in Melbourne who are not identified as eligible and hence are not formally introduced to the MCH service.

### Refugee mentor program

The *Refugee Family Mentoring and Resource Program* involves ‘mentors’ from Assyrian/Chaldean, Karen/Burmese and South Sudanese backgrounds working directly with families to support their access to early childhood services including MCH services, kindergartens, early intervention and family welfare services [45]. This model supports families through organised playgroups [46] and operates differently for each cultural group to meet the needs and circumstances of the playgroup participants.

At two Karen playgroups the refugee mentor was effective in introducing parents who arrive with pre-school aged children to the MCH service. The refugee mentor’s role involves organising mothers and children to attend their ‘key ages and stages’ visits and working closely with the MCH nurse to ensure mothers are seen in this group setting. This model is working so well that parents are ‘referred’, usually by healthcare providers, to the playgroup rather than to the MCH service itself.

It was evident that the model at the Karen playgroups enhanced access to the MCH service for this group of people and likely that the group setting provided an opportunity for culturally supportive discussions about child health and development. However, consideration must also be given to ensuring that women have access to individual appointments, with an interpreter, where they may feel less constrained about discussing issues of concern with the MCH nurse. When the bilingual community workers and the managers were asked if the women attending group sessions would be receiving the same level of care as an English-speaking mother with an individual appointment it was reported that they would be getting the same level of care because of the skills of the MCH nurse in being alert to broader issues:

"*…the nurse might look like she’s just weighing and measuring the baby but they’re on top of everything, they know, they’re watching the interaction, so even if that’s not verbal, if there are concerns the nurse does pick up on them (Manager of community workers)*"

Several participants from Iraqi and Assyrian Chaldean backgrounds were not linked to a refugee mentor and had not heard of the MCH service. One Iraqi participant had heard about the service from her husband who had arrived in Australia prior to her.

### Promoting continued engagement with the MCH service

Many participants across all cultural groups described how important the service was for them. The concepts of preventive health, well-child checks and early detection of child development delays were new for all participants. One participant explained the value of the MCH service for her and her community:

"*It is very, very useful and important for us because each time we go back we talk to maternal and child health and they ask the question like okay, if your child is five months old this is how much he weighs and that’s how much he grows. She show us a graph and the proportions and everything and then say this year your child’s able to do that at this stage - so we know the child’s development, very useful for me to know that. (Karen FG)*"

The ‘refugee mentor’ model functioned differently for the Assyrian Chaldean community. The women in this group had been in the country longer and could understand basic spoken English but were not confident in speaking it. The refugee mentor encouraged mothers to make their own appointments at the MCH centre. This was working well for some although several had not heard of the service before. Several Iraqi mothers reported that they didn’t realise until they used the service that the nurse also checked the mother’s physical and emotional health, they described this as a new concept and felt it was important to them.

"*She had all three children here in Australia, and always the nurse was following her up, even her emotional [health], every month. She followed the children's health and mum's health too and always if she couldn't visit or a different nurse [couldn’t visit], the nurse would ring her and ask her about her emotional [health] and how she's feeling…but she's surprised because in Iraq, in her country, she didn't hear about that, like straight away [the MCH nurse would follow-up and check] they were feeling okay after they had their babies. (Assyrian Chaldean FG)*"

Again, the ‘refugee mentor’ model worked differently for the South Sudanese mothers. This group had not identified having the MCH nurse visit their playgroup as a priority. Although there were some mothers with children born in Melbourne who were using the service, others had used it previously but had encountered significant barriers for continued utilisation of the service. One mother reported that she had walked to a MCH centre with her children and was given a phone number and told to return home and call to make an appointment. Another mother had a new baby and was told that her preferred centre, where she already took her other children, was too busy and that she would have to go to another one which was further away from her home and difficult for her to walk to with her multiple children. She did not want to question the direction to attend another centre because she didn’t want to appear as though she did not care about her children.

"*…I ask them why you send me to a place I don’t know? I can’t talk or I can’t make complaint because it’s for my health my baby…I have to go, I don’t have a choice. I am the one who care about my baby. So now I have to walk very far. (South Sudanese FG)*"

### Voluntary nature of MCH service

Several MCH nurses reported that it was often difficult for them to call parents to remind them that they were due for an appointment aligned with the ‘key ages and stages’ visits while also trying to explain that the service is free and completely voluntary. Some mothers reported that they would like to see the nurses more often than the 10 ‘key ages and stages’ with the gap between 2 and 3.5 years in particular, considered too long. Most parents were unaware that they could access the MCH service for individual consultations even if it was outside of the ‘key ages and stages’ visits. However, the MCH nurses in one area reported that although they would welcome people approaching them, they did not actively encourage it, as they were extremely busy and felt they did not have the capacity for additional appointments. When mothers were asked where they would seek information on their child’s health and development, should they ever need it, responses included: family, friends and their General Practitioner (GP), rarely was the MCH nurse viewed as a source of information.

The refugee health nurses in one area explained that although their service is similar in that it is free and voluntary to use, they employ an Arabic Liaison worker to call families to make appointments. They found that this helped to promote understanding of their service and they also reported that they had few cancellations or appointments that needed to be rescheduled. They felt that if families received a phone call from someone who spoke their language it made it more personal and therefore they were more likely to trust the service and allow the nurses to visit them in their home.

### Transport difficulties

Participants identified transport difficulties in getting to the MCH centre. Although some women could walk to their closest MCH centre, others reported that they had no access to private transport, that public transport was difficult to use as it was not close their home or problematic to use while managing several young children, including walking toddlers and infants in prams. Participants reported that at times public transport was unreliable, making it difficult to get to appointments on time. One refugee health nurse reported how she oriented her appointment times to reflect the local bus timetable that many of her clients used.

### Continuity promotes engagement

Building a relationship between the nurse and client was reported as critical for building trust and for continued engagement in the service, with consistency between nurse, interpreter and client preferred. This was identified as a key component of trusting and productive relationships to support child health and development and maternal wellbeing. One MCH nurse reported that it took her one year of developing a trusting relationship with a mother of refugee background before the mother disclosed her violent situation at home with her partner.

"*Continuity of staff is another thing, because if you see, every time you come, if you have a problem and you see a different person you're going to disengage with the service because you don't want to be going over the same things. Yes, I do have mums saying "I don't want to have to repeat this over and over." (MCH nurse)*"

### Language challenges

Most women spoke very little English and were not studying English as they were involved in full-time child rearing. Key language barriers impacting on MCH service use and described below included: working with interpreters, using telephones, appointment reminders, access to translated information and working with bilingual staff.

### Working with interpreters

There were mixed experiences across the different locations regarding ease of access to interpreters. Some of the healthcare professionals reported that interpreters often relied on public transport to get to appointments which was, at times, unreliable, while others were unwilling to travel long distances to get to MCH centres or people’s homes. For both MCH nurses and participants, there was a preference for in-person interpreters. Telephone interpreters were used when necessary, although they were reported as problematic (mobile phones cutting out, telephones with no loud-speaker/hands free option or limited volume). Health professionals reported that using the same interpreter with the same clients assisted with developing a good relationship with the family.

"*The phone interpreter is too impersonal. And I found that a lot of them use mobile phones so you're constantly cutting out. You don't know who this person is. And if you end up using the same interpreters on a regular basis then the mothers get used to the interpreters and vice versa and you can build a really nice relationship. (MCH nurse)*"

The length of appointment time was also reported by health professionals as not long enough even though they were allocated extra time to work with an interpreter. They stated that often there were complex or multiple issues that needed to be considered and there was not enough time to do everything that was required.

Participants reported that interpreters were not always used at appointments and that they often relied on using body language and facial expressions to communicate. Some participants, mostly the Karen and South Sudanese reported that they replied ‘yes’ to the health professional even if they didn’t fully understand what was being asked.

"*Q: When you go to the maternal and child health nurse is there an interpreter there?*"

"*A: No, just show, do the body language*"

"*Q: Body language yeah, so you can’t really ask any questions?*"

"*A: No, we just say yes, yes*"

"*Q: Has there been an opportunity to use a phone interpreter?*"

"*A: We just need to go and have our immunisation done so maybe we don’t need the interpreter (Karen Focus Group)*"

Several Bhutanese, Iraqi and Assyrian Chaldean participants reported that when they felt it was important for them to have an interpreter they asked for one because they did not want to misunderstand any health–related information that might compromise their child’s health.

### Using telephones

Several health professionals reported that having a central telephone line to the MCH service in their area worked really well for them to call and make appointments for their clients. However, this method was not favoured by mothers who preferred to directly call a familiar nurse to make an appointment. The following examples demonstrate the challenge for refugee background families to make appointments using telephones.

"*A: Actually I haven't been to the maternity nurse with my son that much because I was very busy with my parents and my father was very sick and I was the only one who was taking care of him. So I didn't have a chance to take him to the nurse.*"

"*Q: Would you ever feel like you could call the nurse to ask her [about her concern]?*"

"*A: I called one time, but when I called her she told me I have to call [the City Council] because they are the ones who make, arrange an appointment with you. So when I called [the] City Council, the time they gave me [an] appointment but this time I'm very busy, when its school time, when I was with my dad at the hospital… (Assyrian Chaldean FG)*"

In another example, a mother knew she had to make an appointment for her child to see the nurse; however it took her three days to build up the confidence to pick up the phone and ring to make an appointment. The mother explained that she knew she would have to leave a voicemail and was not confident that her English was good enough for someone to be able to understand her. She called and left a message but realised a few days later that she didn’t leave a phone number for the nurse to return her call and had to repeat the process.

"*…a lot of problem when you leave a message on the phone. I think it’s good for some people, they leave the message and they call them back but some other people they are afraid. They don’t know English and how they go on the [answering] machine. For me, it took me 3 days to make an appointment myself, I got the card, the number and I got the phone but I can’t…because it scares me. (South Sudanese FG)*"

In Victoria, there is a free MCH advisory service phone number available 24 hours, 7 days a week for the public to use. The majority of participants had not heard of this service before. For the few participants who had attempted to use it, they had found the telephone number in the MCH child health record book but had little success using it. They reported that accessing an interpreter took too long and instead went directly to the local hospital.

### Importance of reminders

Mothers explained that it was easy for them to forget appointments that were made in advance and those that received reminder calls were appreciative of them and felt this assisted with keeping scheduled appointments. Mothers who had not received reminder calls reported that this would be helpful to them.

*And you write it somewhere on the paper if you forgot, its hard sometimes to remember where and when is your appointment…they should call you to remind you[if] they miss the appointment because they can’t, they forget to write [the appointment information] in the [child health record]book and they [the mother] forget to call back to make another appointment. (South Sudanese FG)*

Some health professionals also reported that providing a map or instructions for how to get to the MCH centre was very useful, because often parents were confused between hospitals, GP clinics, community health centres and MCH centres – even if they had been to the MCH centre previously.

### Access to translated information

In contrast to those groups supported by a refugee mentor, a group of Karen participants had minimal engagement with the MCH service. Although they all reported visiting their local MCH centre at least once, and had an appointment with the nurse, they spoke about the frustration of knowing that the child health information available in the waiting room was important but that they could not read it.

"*Q: Is it useful going to the maternal and child health nurse? Do you find that you get the information that you want?*"

"*A: If we can [understand] English it would be very beneficial because there is a lot of information on the walls*"

"*Q: Would you like to be able to read in English or would you like that information in Karen?*"

"*A: Yeah if someone comes and gives us information this would be good and talks in Karen yeah (Karen FG)*"

Mothers also reported receiving referral letters in English, for example a referral from a GP to a specialist, and not only having to find someone to read the letter but not understanding how or where to go to send letters by facsimile. MCH staff also noted the lack of follow-up in relation to referrals. They reported that many refugee children required specialist healthcare and were concerned that this care was not being delivered as a failure of the referral process.

### Working with bilingual staff

The Karen bilingual worker was also an accredited interpreter and she facilitated the group appointments at the playgroup and this worked well for participants and the nurses. It was reported that because she could assist with not only language, but also aid in cultural understanding, that the nurses felt much more confident in working with that particular community. It is Victorian government policy that all healthcare providers work with an accredited interpreter at appointments with non-English speaking clients; this has implications as not all bilingual workers (who are often trusted by the community) have interpreter accreditation. Several nurses reported the difficulties with engaging clients from African countries. The South Sudanese refugee mentor also reported these difficulties, as they persisted even though she had attempted to resolve these using similar strategies to the Karen refugee mentor. This still remains unclear and requires further exploration.

### What is working well and what could be done better

When asked about their experience of using the MCH service, all community participants who had used the MCH service spoke highly of the service they received and had no complaints. Participants’ comments implied that the health professionals ‘knew best’ and so they did not question the service they received or have any suggestions for service improvement. A Karen mother explained:

"*For maternal and child health they are higher than us, how can we give them advice? They already understand everything…because we tell them our problem and they answer our problem. (Karen FG)*"

### Supporting MCH nurses

The MCH nurses spoke about fulfilling multiple settlement roles outside their scope of practice, such as that of social workers or case managers. This occurred because they had developed a trusting relationship with the mother who then felt she could help her with other issues. The MCH nurses reported that they would like to be better informed about other services and programs being delivered to refugees to ensure they are complementing one another rather than duplicating or ‘undoing’ the work being done by others. Furthermore, the MCH nurses and other healthcare professionals reported concerns about the wellbeing of refugee background people without any family or community support, highlighting that there are some people that are ultimately even more vulnerable and requiring support. The MCH and refugee health nurses suggested that it would also be useful for them to know what programs and support services were available so that they could link their clients to them rather than feeling as though they are leaving them isolated.

"*They’re the ones you worry about because they are doing it tough. (Refugee health nurse)*"

The MCH nurses reported that they had recently participated in ‘cultural competency’ training; however, it did not include a specific focus on working with refugee clients. As mentioned previously, some MCH nurses reported difficulty engaging with clients of African backgrounds and felt that culturally specific training would be helpful.

### Increasing home visits

Providing home visits was seen as critically important for continued engagement with the MCH service. The ‘enhanced’ stream of the universal MCH service responds to the needs of vulnerable children and families at risk of poor outcomes, in particular where there are multiple risk factors [47]. Most families of refugee background would be considered ‘vulnerable’ however, it was not evident that they had been offered or were currently receiving this service. Although mothers did not explicitly report that they would prefer home visits (as they may not have known that this service was available to them) it was clear that continued utilisation of the service needed to be made easier for them. The capacity for MCH nurses to provide home visits whether by the universal or the enhanced service, varied across the different areas. Where MCH nurses were conducting home visits, for example with families with several young children and limited access to private transport, the nurses reported that this assisted with establishing an on ongoing relationship that builds trust to support parents’ retention in the service. Perceptions of personal safety were also identified by healthcare professionals as reasons for clients preferring home visits compared to walking or catching public transport to MCH centres. This was not in relation to feeling unsafe, being harmed or living in an unsafe neighbourhood, but fear of the ‘big unknown’ that comes with moving to a new and unfamiliar area.

### Improved client records

The MCH nurses reported that they kept their own personal records of client information – rather than centrally located databases. In one local government area, they were able to produce a list of the languages other than English spoken by new mothers enrolled at the MCH service. The MCH nurses explained that they would not easily be able to identify from their client databases whether their clients were of refugee backgrounds. The MCH nurses reported that the information they collect from new clients does not always include the mother’s (or child’s) country of birth or their year of arrival to Australia. They all reported that this is something they would like to be able to do in order to have a better understanding of who their clients are and that it wouldn’t take much more additional time to complete this as part of their record keeping for clients.

### Co-location of services

To meet the many needs of refugee clients (as well as other vulnerable parents) there was strong support from the MCH nurses and healthcare professionals for the co-location of early childhood, social support and English language services.

"*…because if you have the playgroup working with the Maternal and Child Health in the same building you need multi-faceted buildings, so you could have English classes. So if you've got…a hub, a population, you need to have the English classes in the same building rather than have to have them go somewhere else. One stop shopping, if we can do that they will manage a lot easier. So if they need counselling they can meet the counsellor, so they're not going off to some strange place to see a counsellor to talk about all their traumas, they can actually know that this person they've met, they've seen and they're already in the same building. (MCH nurse)*"

## Discussion

This study has documented and synthesised the perceptions and experiences of MCH services in Australia for people of refugee backgrounds and their service providers. With 18 service provider participants and 87 community participants from several different cultural groups, this study has been able to appropriately engage with participants typically described as ‘hard to reach’ and therefore hard to ‘research’. A cultural competence approach was used to underpin this study with various community involvement at all stages of the research process.

Cultural differences, language difficulties, lack of awareness of available services, and lack of health provider understanding of the complex health concerns of refugees can all contribute to inhibited access to healthcare. The findings indicate that the issues affecting initial and continued access to the MCH service by people of refugee backgrounds are multifaceted and arise from socioeconomic disadvantage (e.g. access to private transport), pre-arrival experiences, and differences in language and culture. These demonstrate the importance of considering the results within a socioecological framework in order to determine what multi-level adjustments to the MCH service might be effective in promoting improved and continued access for families of refugee backgrounds

The results suggest that families who have children born in Melbourne have reasonable initial access to MCH services, however, a clear gap in settlement services was identified in relation to linking parents who arrive in Australia with young children to the MCH service. The success of this linking process depends on the ability of the sponsor of the family; the knowledge of the community guide/case worker; or simply whether the information is provided to the newly arrived family at a time when it can be retained, given all the other demands and stresses associated with settlement. Although this issue has been identified previously [13] the gap remains, with no strategic, coordinated or formal mechanism for on-arrival settlement services to identify families with young children and link them systematically with their local MCH service. Given that this service has a significant role in early detection of developmental delays and health problems for children and mothers, it is critical that this is addressed.

A significant barrier for continued engagement, with not only the MCH service but any other service for mothers of refugee-background is a low-level of English proficiency. For those who could understand spoken English, most were not confident in speaking English especially to strangers, over the phone or leaving voicemail messages. Most parents reported wanting to learn and practice English, although many were not studying due to full-time child rearing roles. The MCH service needs to be mindful of the language difficulties experienced by refugee backgrounds mothers and respond to these service access barriers appropriately. There needs to be alternative ways in which mothers who are not confident in using telephones and leaving voicemail messages can make appointments. Bilingual community workers or interpreters could assist in this way by telephoning parents to book them in for their appointments and helping them with either community transport or organising how they are going to travel to get to their appointment. Guidelines have been developed to support organisations and the bilingual workers they employ [48]. Group visits should also be encouraged; however, MCH nurses need to allow for mothers to comfortably raise any issues of concern with them; this may be achieved by making individual appointments with those mothers, facilitated by an interpreter or bilingual community worker, where they exist, or by providing each mother with an opportunity to meet the nurse in a private room during the group visit. For longer term solutions, extending the co-location of MCH and other social and health services with flexible English language classes may be a useful means of supporting access to these and other services and promoting positive settlement for families of refugee backgrounds.

Participants reported their experience of using MCH services as positive, though participants may have withheld information in the focus group setting because they did not wish to disclose negative experiences in front of their peers, the researchers, their community representatives/bicultural worker, or the interpreters. Bandyopadhyay and colleagues in their study of birthing experiences in Australia suggest that immigrant women (including but not exclusively refugee background women) view their local community as ‘very mother and baby friendly’ compared to women proficient in English. The authors suggest this might be because they find that the services and supports are better than in their country of origin or that immigrant mothers do not want to be critical of their new country or that they simply have lower expectations of community services and supports [49]. These possibilities are also plausible in this study.

Although other studies [6] have identified lack of finances as a constraint for people of refugee backgrounds to access health services, this was not identified as an issue as MCH services in Victoria are provided for free. However, access to private transport and efficient, reliable and accessible public transport, were reported as significant barriers for parents to access health services, particularly for arriving on-time at scheduled appointments. MCH nurses reported the need for being flexible in their services delivery times and often making appointments that reflected local bus timetables. Home visits by professionals are used widely as a strategy to provide support to families with identified vulnerabilities or risk factors [15]. Increasing MCH nurses’ ability to conduct home visits would support ongoing and long-term retention of families in the service. A systematic review revealed that for women and families at high risk for either family dysfunction or postpartum depression, home visitation by a nurse, resulted in a reduction in depression scores (as measured by the Edinburgh Postnatal Depression Scale) [50]. Such home visits need to be conducted in a sensitive manner, given that previous Canadian research, although not with culturally diverse participants, has demonstrated that mothers receiving home visits felt the nurse was ‘watching over them’ which created a sense of fear and a lack of trust [51]. This was not raised as an issue in this study with refugee background women. However, it does highlight the importance of effective engagement that is sensitive, including an appropriate interpreter, in order for a good relationship to be formed, to not exacerbate any feelings of vulnerability. This is supported by recent Australian research conducted to promote improved maternity care for women of refugee backgrounds [52].

The study findings also revealed that the different levels of health service entitlements by visa category may further complicate the provision and use of MCH services for providers and clients, though this may now improve in light of recent policy changes in this area, as previously mentioned. This has been previously reported by Davidson and colleagues as an area of concern requiring attention [6]. The healthcare professionals were concerned about those arriving on ‘sponsored’ visas whereby the sponsor themselves would also be considered vulnerable and not in a position to be supporting newly arrived people in accessing needed services. Furthermore, those identified as arriving alone and considered to have ‘refugee-like’ experiences were also of concern to healthcare professionals. Critical here is for the MCH service to be able to identify whether clients are of refugee background in order to tailor their services appropriately. As stated previously, a recent state-wide report examining the health status of refugee children and young people reports that a range of data collection systems are used by MCH services and that although maternal country of birth is recorded and could be used as a proxy for determining refugee background, it is not possible to extract and use this data [17]. It is recommended that state-wide client records are established and routinely collected so that refugee background clients can be identified and their ongoing retention in the MCH service monitored.

Internationally, community-wide interventions have utilised universal health services to improve preventative services for children and families, though not necessarily refugee background families. In the United States Margolis and colleagues conducted a large observational intervention study that aimed to achieve changes in the delivery of health care, particularly the interaction between those providing the care and low-income pregnant mothers to improve health and developmental outcomes for children [53]. Health care staff received training, support and supervision; there were structured protocols for care delivery, and regular feedback data about implementation of the program. The authors reported high levels of family participation in the services, changes in the delivery system, and improvements in preventive health outcomes. Intervention group women were significantly more likely to use contraceptives, not smoke tobacco, and have a safe and stimulating home environment for their children. Children were more likely to have had an appropriate number of well-child care visits and were less likely to be injured. The authors also reported that many improvements continued to be seen since project completion. From this study it was concluded that multi-level, interrelated interventions directed at an entire population of mothers and children hold promise to improve the effectiveness and outcomes of health care for families and children.

A national study conducted in the United States investigating parent satisfaction with well-child care for young children, reported that Hispanic parents, particularly those responding to a survey in Spanish (rather than English) gave lower satisfaction ratings [54]. Although, overall, parents generally reported high levels of satisfaction with well-child care visits, the authors suggested that it is important to understand how parent perceptions of time adequacy and their ability to ask questions about topics that are important to them, relate to the amount of information that health care providers are able to convey. The authors suggest that allocating more time for appointments is appropriate given that the number of recommended topics to cover in each appointment has increased over the years [54]. It is also reported that health care providers use the Parent Evaluation of Developmental Status (PEDS) to help them provide more responsive and targeted care and information on issues that are of concern to parents [54]. In Victoria, the MCH nurses are already required to complete this instrument with parents as part of the 10 ‘key ages and stages’ assessments, however, it is not known how appropriate and relevant this tool is for families of refugee backgrounds. Furthermore, our findings indicated that mothers of refugee backgrounds would like to see the same MCH nurse at their appointments, in the United States is has been demonstrated that having a regular clinician is associated with greater volume of preventive visits for children and is associated with fewer emergency department visits and hospitalisations and more frequent parent reports of discussing specific health topics [55]. However, visit frequency and continuity of clinician shows minimal impact on timeliness of preventative care and adherence to recommended guidelines. Inkelas and colleagues report that in the United States, for young children, having a regular clinician yields the greatest interpersonal quality gains in community health clinics and for African American and Hispanic children in contrast to non-Hispanic white children and they suggest that having a regular clinician may narrow the disparities gap between racial and ethnic population subgroups witnessed in primary health care [55]. Furthermore, programs and policies to improve parental education, health literacy, the quality of service provider communication and quality improvement strategies for health care systems are critical to improve ‘family-centred’ care and have the potential to reduce racial and ethnic health disparities [56]. Although, it is also acknowledged that service provider training and supporting clinicians to promote high quality patient interactions for patients who have varying levels of education, health literacy and English proficiency are keys areas requiring further attention [56].

Innovation, flexibility, and culturally competent service models appear to be critical for ensuring that services interact with the contexts and experiences of refugee families to optimise their start in a new country. There are different models of service delivery that showed promise in terms of improved access to MCH services. These include the use of bilingual workers/refugee mentors, utilisation of playgroups to build service awareness and engagement and social support, strategies to promote self efficacy, group appointments, and co-location of services. The ‘refugee mentor’ model was working well for promoting MCH group appointments with Karen families, however, this is provided they are attending playgroup in the first instance. Research is needed here to assess whether this model is likely to enhance or hinder mothers’ capacity to access services for themselves as they become more settled. Furthermore, while this was reported to be a successful model by both providers and users of the service for promoting access to the MCH service, there is the potential for this to be a limited service – with each parent having less opportunity to discuss any individual concerns – compared with the individualised service received by English speaking parents. Similar concerns exist for clients when no interpreter is used – i.e. even though the MCH nurses may be able to “assess more than parents realise”, the parent does not have the opportunity to discuss any particular concerns that may not have been evident to the nurse, for instance intimate partner violence. In developing a model of best practice for refugee maternity care, Correa-Velez also advocated for continuity of care, quality interpreter services, educational strategies for both women and healthcare professionals and the provision of psychosocial support to women from refugee backgrounds [52]. Learnings from our research suggest that there may not be one ‘model’ of best practice for promoting maternal and child health for refugee background families, but a suite of strategies that are flexible and adaptable and are reflective of the clients’ cultures, languages, existing social groups and resources of local service providers – both mainstream and culturally-specific.

This research has identified a range of holistic strategies to improve access and engagement with refugee background families, however, in many cases the options are limited by the need for efficient use of scarce resources. In other cases, such as the introduction of a central telephone booking service, we have demonstrated that such efforts which have aimed to improve access for many are actually counter to the expectations and needs of the clients. Participants faced extreme difficulties in terms of having the confidence to use telephones to make appointments, particularly when they were required to leave voicemail messages. MCH services could proactively work in partnership with bilingual community workers to call clients directly to make appointments. Where these workers are not available, interpreters could also be utilised for this purpose. In many cases, it was the role of the ‘refugee mentors’ to initiate service access for parents of refugee backgrounds and to enhance the cultural knowledge and capacity of healthcare professionals and their organisations. The MCH service provides an opportunity to focus on the dynamics between parents and children and to locate the issues facing refugees in their wider family and community context. Given the social isolation and lack of knowledge of services and systems (for example, education as well as health) for refugee background families, MCH services also potentially play a vital role in linking families to communities and services more broadly. The role played by bicultural workers should be recognised and utilised in a way that benefits clients and service providers. This, of course, is only possible in areas with high prevalence of a particular language. Therefore, alternative models for client engagement should be trialled. One such model could be a ‘drop in’ service whereby an allocated day and time could be promoted to come to the MCH service with an interpreter available for that time. If large numbers were to arrive at the time period, it would provide an opportunity for other healthcare providers to attend to meet the clients to introduce themselves and their service, as well as an opportunity to conduct community health promotion sessions on particular topics. Another model to be trialled would be MCH nurses attending at venues where parents already gather, such as playgroups, kindergartens and English language centres, to conduct group information sessions that promote the MCH service, provide health information and enable that first initial trust-building contact that could lead to parents wanting to make individual appointments at the MCH centre with a nurse who is now familiar to them. The importance of building trusting interpersonal relationships between nurse and client is well established and Jack and colleagues report that the quality of these relationships should be continuously assessed and perspectives of mother’s satisfaction of these relationships sought [51].

Meaningful engagement with refugee families is critical for sustainable health service utilisation. If this does not occur it can ultimately further isolate and marginalise refugee families with infants and young children. To promote the responsiveness of the MCH service to the needs of people of refugee backgrounds requires awareness of the diversity of that population in key aspects such as culture and experiences prior to arrival and in the course of settlement. The ‘refugee mentor’ model described here is one that has the potential to promote effective communication and cooperation, and avoid cultural ‘blind spots’ and can lead to a progressive depth of understanding between the MCH nurse (or any health service provider) and the client. This will hopefully and ultimately improve referral to specialist care where required, clinical diagnosis, and health and psychosocial outcomes for refugee background children and families.

The study was undertaken in two areas of outer urban Melbourne and the findings may not be applicable to other locations in Victoria (e.g. rural and regional areas) or more generally. The sampling frame includes multiple cultural groups across several different settings but there may be sociocultural issues that were not uncovered in this study that apply to other groups.

## Conclusion

There were several modes of initial access to MCH services in Melbourne identified for families of refugee backgrounds. However, families who arrived in Australia with young children had limited opportunities for utilising the service and there were significant barriers affecting continued engagement with the MCH service for all families. Understanding the complexities of a new healthcare system and establishing a relationship with the MCH service is difficult, and is exacerbated by the challenging circumstances of settlement. There also needs to be formal processes between settlement programs and MCH services to link families arriving with young children to the MCH service. This highlights the support needed from bilingual/bicultural workers to bridge the language and cultural gap that is paramount for refugee clients and service providers. Provision of refugee focussed training for service providers and a strategically coordinated approach is likely to facilitate access, build rapport and ongoing engagement and retention to the service for families of refugee background. Innovative culturally competent strategies to organise individual MCH service appointments should be trialled and evaluated to develop a MCH system that promotes refugee maternal and child health.

## Abbreviations

CALD, Culturally and Linguistically Diverse; DEECD, Department of Education and Early Childhood Development; MCH, Maternal and Child Health.

## Competing interests

The authors declare that they have no competing interests.

## Authors’ contribution

All authors 1) have made substantial contributions to conception and design, or acquisition of data, or analysis and interpretation of data; 2) have been involved in drafting the manuscript or revising it critically for important intellectual content; and 3) have given final approval of the version to be published.

## Pre-publication history

The pre-publication history for this paper can be accessed here:

http://www.biomedcentral.com/1472-6963/12/117/prepub
